# Supplementary material for: The Activation of Nrf2/HO-1 by 8-Epi-7-deoxyloganic Acid Attenuates Inflammatory Symptoms through the Suppression of the MAPK/NF-κB Signaling Cascade in In Vitro and In Vivo Models
Source: Antioxidants (Basel). 2022 Sep 7;11(9):1765. doi: 10.3390/antiox11091765 (PMC9495988; doi:10.3390/antiox11091765)
Supplement: Supplementary file 1 [file antioxidants-11-01765-s001.zip › antioxidants-1880944-supplementary.pdf]

Activation of Nrf2/HO-1 by 8-Epi-7-deoxyloganic acid attenuates inflammatory symptoms through suppression of MAPK/NF-κB signaling cascade *in vitro* and *in vivo* model

Shakina Yesmin Simu[1] , Md Badrul Alam[2,3] and Sun Yeou Kim[1*]

[1]College of Pharmacy Gachon University, No.191, Hambakmoero, yeonsu-gu, Incheon 21936, Korea

[2]Department of Food Science and Biotechnology, Graduate school, Kyungpook National University, Daegu 41566, Korea

[3]Food and Bio-Industry Research Institute, Inner Beauty / Antiaging Center, Kyungpook National Uni-versity, Daegu 41566, Korea

*Correspondence: Sun Yeou Kim (S.Y.K.); Tel.:+82-102292-9232; email: sunnykim@gachon.ac.kr

Table S1: List of the primary antibodies used in the study

Name	Catalog no.	Company	Antigen	Host
Anti-iNOS	MAB9502	R&D systems	iNOS	Mouse
Anti-COX2	AF4198	R&D systems	COX2	Goat
Anti-HO-1	BS90659	Bioworld Technology, Inc.	HO-1	Rabbit
Anti Nrf2	BS1258	Bioworld Technology, Inc.	Nrf2	Rabbit
Anti-IκB-α	ab7217	Abcam plc	IκB-α	Mouse
Anti-NF-κB (p65)	BS1254	Bioworld Technology, Inc.	NF-κB	Rabbit
Anti-p-NF-κB (p-p65)	BS4137	Bioworld Technology, Inc.	NF-κB	Rabbit
Anti-p-IKKα/β	BS4236	Bioworld Technology, Inc	IKKα/β	Rabbit
Anti-IKKα/β	BS1756	Bioworld Technology, Inc.	IKKα/β	Rabbit
Anti-p-p38	BS4635	Bioworld Technology, Inc.	p38	Rabbit
Anti-p38	BS3567	Bioworld Technology, Inc.	p38	Rabbit
Anti-p-ERK1/2	BS5016	Bioworld Technology, Inc.	ERK	Rabbit
Anti-ERK1/2	BS 6472	Bioworld Technology, Inc.	ERK	Rabbit
Anti-Keap1	BS6783	Bioworld Technology, Inc.	Keap-1	Rabbit
Anti-β-actin	BS6007M	Bioworld Technology, Inc.	Actin	Rabbit
Anti-Lamin B	BS3547	Bioworld Technology, Inc.	Lamin B	Rabbit

Table S2: List of the primer sets used in the study

Gene name		***Sequences***
MCP-1	*forward*	*AACACAGCTACGAAAACC*
	reverse	*CACAGTATGATGTAACAGT*
TNFα	*forward*	*GGCAGGTCTACTTTGGAGTCA*
	reverse	*ACATTCGAGGCTCCAGTGAAT*
IL-1β	*forward*	*ATGGCAACTGTTCCTGAACTC*
	reverse	*CAGGACAGGTATAGATTCTTT*
IL6	*forward*	*GAGGATACCACTCCCAACAGA*
	reverse	*AAGTGCATCATCGTTGTTCATA*
Gapdh	*forward*	*TTGTGATGGGTGTGAACCAC*
	reverse	*ACACATTGGGGGTAGGAACA*
